# Case Report: Catheter rupture of central venous port devices placed via the right internal jugular vein for chemotherapy in gastrointestinal cancer patients: a four-case series

**DOI:** 10.3389/fonc.2025.1620952

**Published:** 2025-09-03

**Authors:** Toshimitsu Tanaka, Fukahori Masaru, Sachiko Nagasu, Asako Kuhara, Masamichi Koganemaru, Shuichi Tanoue, Fumihiko Fujita, Takumi Kawaguchi, Keisuke Miwa

**Affiliations:** ^1^ Multidisciplinary Treatment Cancer Center, Kurume University Hospital, Kurume, Japan; ^2^ Division of Gastroenterology, Department of Medicine, Kurume University School of Medicine, Kurume, Japan; ^3^ Department of Surgery, Kurume University School of Medicine, Kurume, Japan; ^4^ Department of Radiology, Kurume University School of Medicine, Kurume, Japan

**Keywords:** central venous port, catheter rupture, chemotherapy, complications, gastrointestinal cancer, internal jugular vein

## Abstract

**Background:**

Central venous (CV) ports are extensively employed for the administration of chemotherapy in cancer patients owing to their safety and reliability. The rupture of a CV port catheter is an infrequent occurrence; nonetheless, it can effectuate serious complications if left untreated. In this case series, we present four cases of catheter rupture of CV ports implanted in cancer patients via the right internal jugular vein for chemotherapy administration.

**Results:**

Our series comprised one male and three female patients, with an age range of 41–76 years (median age: 61 years). All CV ports were implanted through the right internal jugular vein using a PowerPort^®^ MRI device and were placed for a median duration of 39 months (range: 17–45 months). In three of four instances, CV ports were inactive at the time of catheter rupture; nevertheless, one study participant continued to use the CV port even after it had ruptured. None of the patients presented with any subjective symptoms at the time of catheter rupture. Two cases were detected via subcutaneous tissue swelling during CV port utilization, and the other two were incidentally detected via imaging. The ruptured catheters were located in the right atrium-right ventricle in two patients and in the superior vena cava and left pulmonary artery in the third and fourth patients, respectively. All four devices were retrieved without complications using a snare catheter.

**Conclusion:**

Catheter rupture of the CV port is a rare but potentially grave complication. Therefore, routine monitoring is required, considering the possibility of catheter rupture if the CV port is retained as an implant for an extended period.

## 1 Introduction

Long-term venous access devices, such as subcutaneously placed central venous (CV) access devices, were first introduced in 1982 and are widely used as venous access for chemotherapy in oncology patients (1). Chemotherapy should be initiated after CV port placement because it allows reliable drug delivery and safely reduces the risk of extravascular invasion with less patient distress, even among patients with difficult peripheral venous access (2, 3). Hence, the widespread use of CV ports has raised the issue of complications associated with their prolonged placement.

The overall complication rate associated with CV port placement has been reported to be 7.2–12.5% (4). Port system infection is the most common complication, followed by catheter-related thrombosis (5). Catheter rupture of the CV port is an infrequent complication, with a frequency of 0.3–2.9% (6, 7). However, in these reports, the CV port was placed via the subclavian vein. Placing a CV port via the internal jugular vein is considered significantly less risky for catheter fracture than placing it via the subclavian vein (8), but this needs to be verified further. Catheter rupture is often asymptomatic but can cause serious complications; hence, removal of the ruptured catheter should always be attempted (6).

Here, we present four case studies of catheter rupture of CV ports implanted via the right internal jugular vein for the administration of chemotherapy in patients with gastrointestinal cancer. The ruptured catheter was retrieved using endovascular technique.

## 2 Case presentation

### 2.1 Case 1

**Figure 1:**
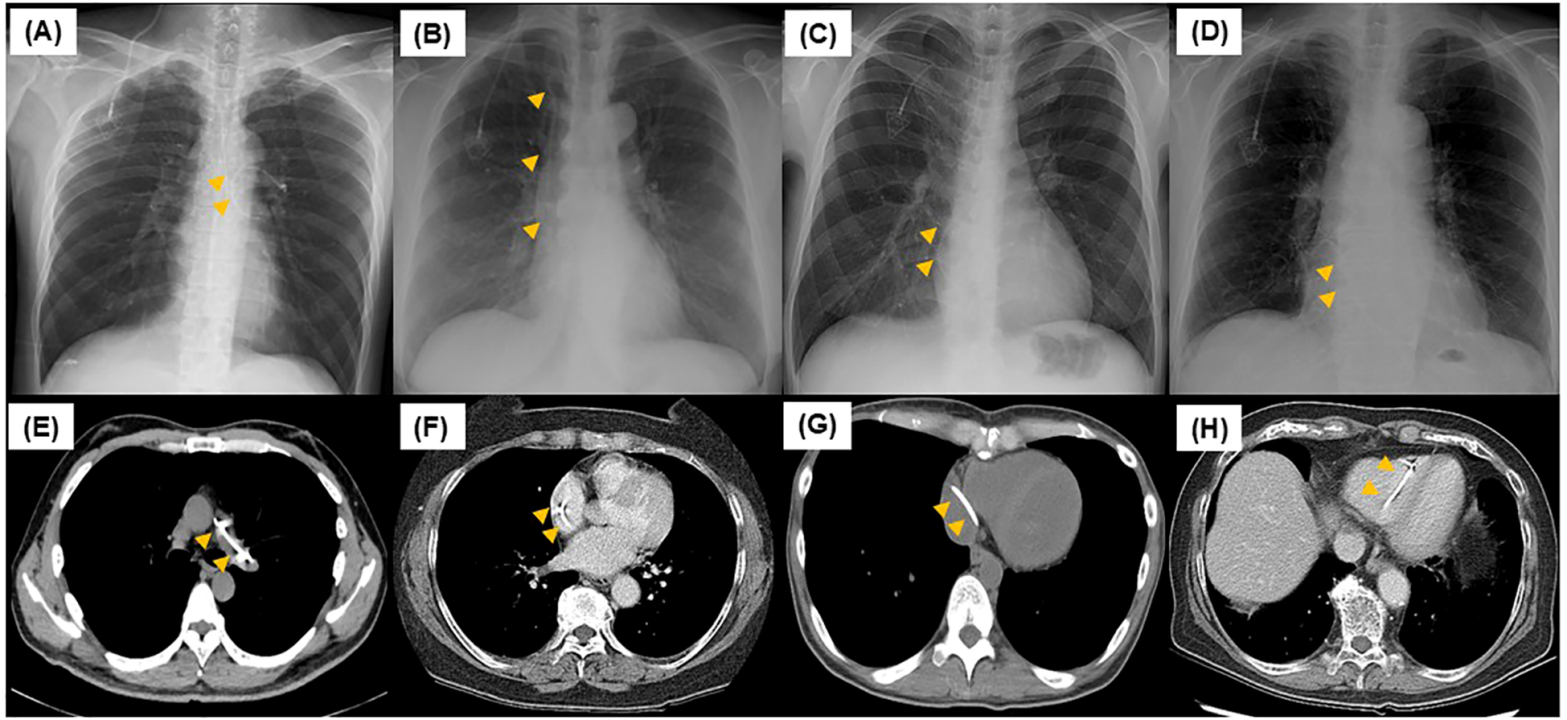
Chest radiography and computed tomography scan at central venous port rupture. For case 1 **(A, E)**, the ruptured catheter was in the left pulmonary artery. For case 2 **(B, F)**, the ruptured catheter was in the superior vena cava. For cases 3 **(C, G)** and 4 **(D, H)**, the ruptured catheter was in the right atrium.

**Figure 2:**
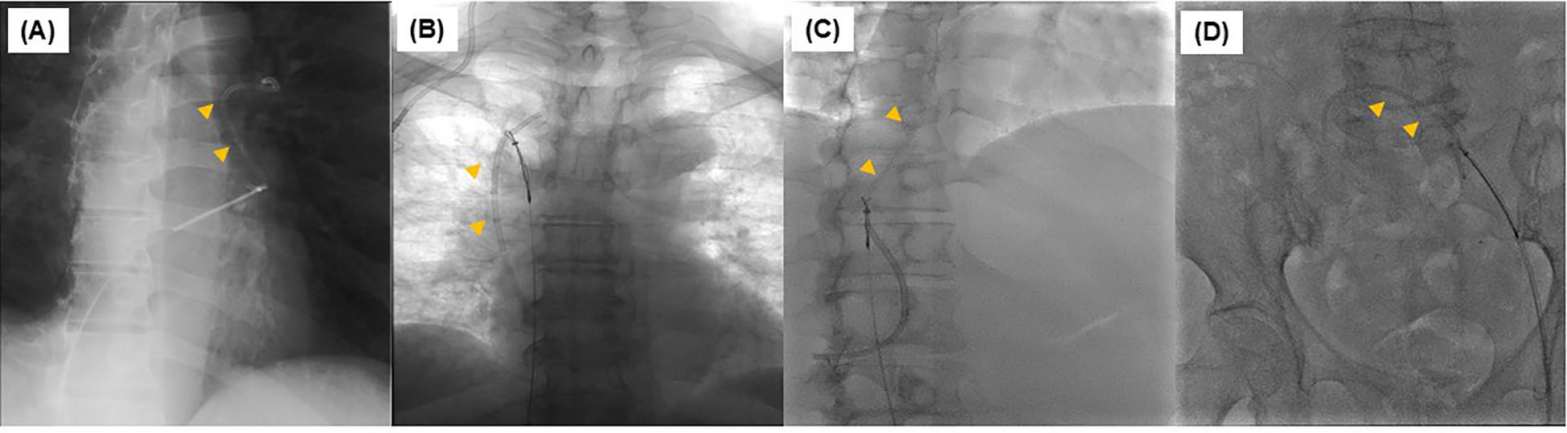
Removal of torn catheter using a snare catheter during an interventional procedure. In all cases from Case 1 to 5 **(A–D)**, all ruptured catheters were removed with a snare catheter.

**Figure 3:**
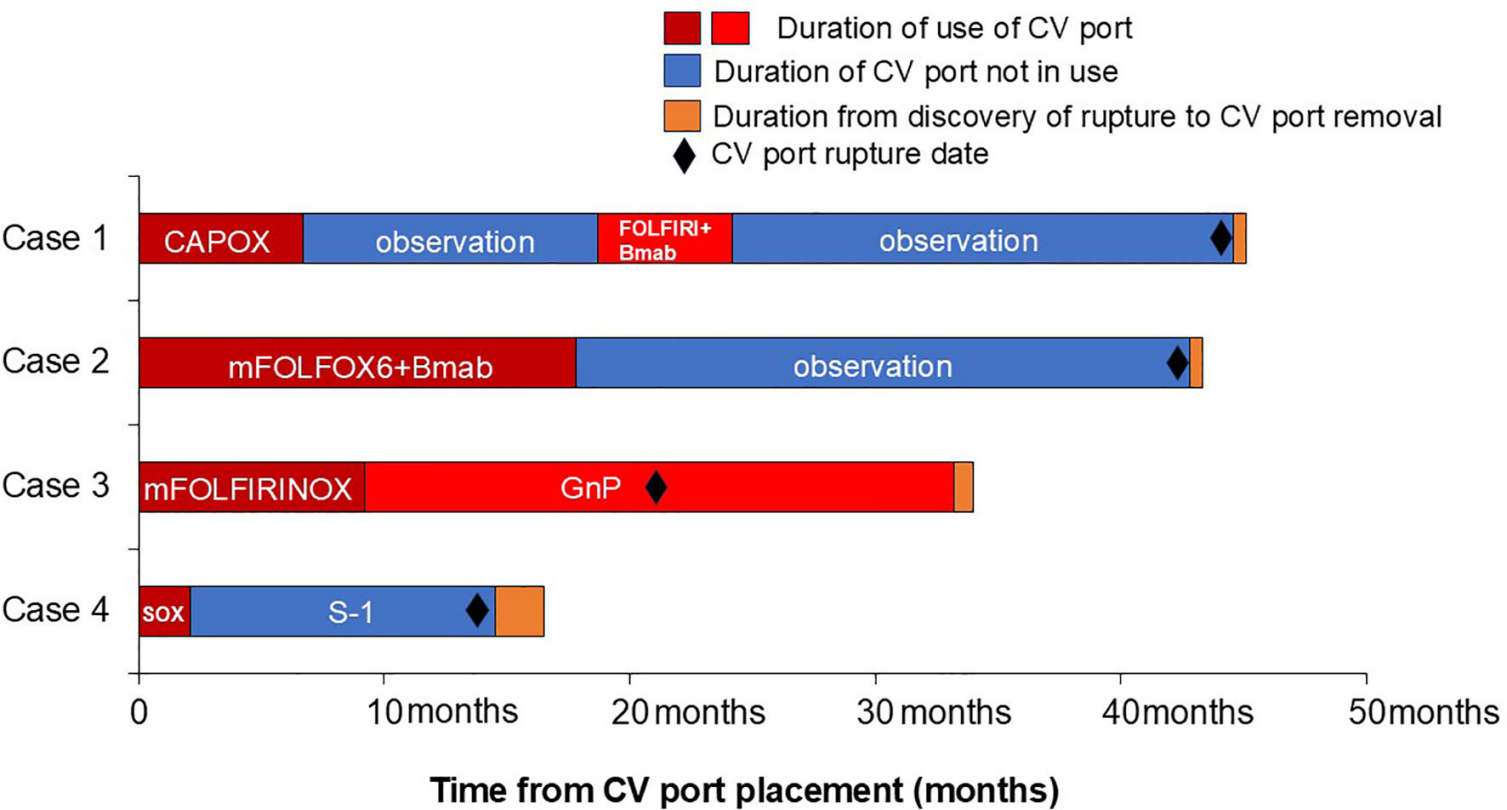
Duration of time between placement of CV port and retrieval of ruptured CV port. CAPOX; capecitabine plus oxaliplatin; FOLFIRI+Bmab; fluorouracil, leucovorin, and irinotecan plus bevacizumab; mFOLFOX6+Bmab; fluorouracil, leucovorin, and oxaliplatin plus bevacizumab; mFOLFIRINOX; fluorouracil, leucovorin, oxaliplatin, and irinotecan; GnP; gemcitabine and nab-paclitaxel; SOX, S-1 plus oxaliplatin.

The patient was a male in his 50s, with a performance status (PS) of 0 (**Table 1**). He underwent laparoscopic anterior rectal resection and D3 lymph node dissection for resectable rectal cancer (pT3pN2M0 pStage IIIb). For postoperative adjuvant therapy, a CV port (PowerPort^®^ MRI; Becton, Dickinson, and Company) was placed via the right internal jugular vein approach. The patient received CAPOX therapy following CV port placement. At the 6-month follow-up, the CV port was still in place. Eighteen months after CV port placement, peritoneal dissemination nodule recurrence was observed in the pelvis, and FOLFIRI plus bevacizumab was initiated for chemotherapy. After approximately 6 months of chemotherapy, the tumor had shrunk, and curative resection was performed (24 months after CV port placement). Forty-four months after CV port placement, peritoneal seeding nodule recurrence was identified on the anterior sacral surface, and subcutaneous swelling was observed once the CV port was used to administer chemotherapy. Catheter rupture of the CV port was suspected, and such rupture was confirmed by chest radiography and computed tomography (CT) findings. The catheter tip was located in the left pulmonary artery; however, no subjective symptoms were observed (**Figures 1A, E**). A snare catheter (EN Snare^®^ 18–30 mm) was used to retrieve the torn catheter (45 months after CV port placement; **Figures 2A**, **3**). No complications were observed. The CV port was then reinserted, and chemotherapy was restarted.

**Table 1:** Patient characteristics.

Central venous port devices placed via the right internal jugular vein in gastrointestinal cancer patients				
	Case 1	Case 2	Case 3	Case 4
Age (years)	50-60	60-70	40-50	70-80
Sex	Male	Female	Female	Female
Primary site	Rectum	Colon	Pancreas	Stomach
Performance status	0	0	1	1
Location of the ruptured catheter	Left pulmonary artery	Superior vena cava	Right atrium	Right atrium
Symptoms at the time of discovery	asymptomatic	asymptomatic	asymptomatic	asymptomatic
Cause of discovery	Subcutaneous swelling	CT image	Subcutaneous swelling	CT image
Duration of CV port placement	45 months	43 months	34 months	17 months

CV, central venous.

### 2.2 Case 2

The patient was a female in her 60s, with a PS of 0 (**Table 1**). She underwent CV port placement via the right internal jugular approach for postoperative recurrence of ascending colon cancer (PowerPort^®^ MRI). Chemotherapy was initiated with mFOLFOX6 plus bevacizumab therapy after CV port placement. Eighteen months after placing the CV port, the tumor was assessed to be in complete response, and she was followed up without further chemotherapy. Forty-four months after CV port placement, CT scan revealed catheter rupture of the CV port. The catheter tip was in the right atrium; however, no subjective symptoms were observed (**Figures 1B, F**). A snare catheter (EN Snare^®^ 18–30 mm) was used to retrieve the torn catheter (43 months after CV port placement; **Figures 2B**, **3**). No complications were observed. The patient was followed up without CV port reinsertion.

### 2.3 Case 3

The patient was a female in her 40s, with a PS of 1 (**Table 1**). She was diagnosed with unresectable pancreatic cancer (cT4N1M1 cStage IV) and underwent CV port placement via the right internal jugular vein approach (PowerPort^®^ MRI). Chemotherapy was initiated with mFOLFIRINOX therapy after CV port placement. Nine months after CV port placement, she had difficulty continuing treatment due to gastrointestinal toxicity, and the treatment was shifted to gemcitabine and nab-paclitaxel therapy. Twenty-one months after CV port placement, a CT scan revealed catheter rupture of the CV port. The catheter tip was located in the right atrium; however, no subjective symptoms were observed (**Figures 1C, G**). Despite catheter rupture of the CV port, chemotherapy was continued using the CV port without rupture detection because the catheter rupture was localized within the central venous tract. Thirty-three months after CV port placement, subcutaneous swelling was observed when the CV port was used, and catheter rupture of the CV port was identified. A snare catheter (Atrieve Vascular Snare Kit, 27–45 mm) was used to retrieve the torn catheter (34 months after CV port placement; **Figures 2C**, **3**). No complications were observed. She continued chemotherapy via peripheral infusion without CV port reinsertion.

### 2.4 Case 4

The patient was a female in her 70s, with a PS of 1 (**Table 1**). She underwent CV port placement via the right internal jugular vein approach (PowerPort^®^ MRI) for recurrent peritoneal dissemination after advanced gastric cancer surgery (pT3N0M0 pStage IIA). Chemotherapy was initiated with SOX therapy after CV port placement. Two months after CV port placement, oxaliplatin was discontinued due to gastrointestinal toxicity and was shifted to oral S1 therapy. Fourteen months after CV port placement, a CT scan revealed catheter rupture of the CV port; the catheter tip was located in the right atrium, but no subjective symptoms were observed (**Figures 1D, H**). A snare catheter (EN Snare^®^ 18–30 mm) was used to retrieve the torn catheter (17 months after CV port placement; **Figures 2D**, **3**). No complications were observed. The patient was followed up without CV port reinsertion.

## 3 Discussion

Long-term placement of CV ports results in complications such as catheter-related bloodstream infections, local infections, thrombosis, and catheter damage. The incidence of catheter rupture of CV ports placed via the subclavian vein is reported to be 0.5–1.5% (9, 10), and the mechanism is thought to be pinch-off syndrome caused by compression between the clavicle and first rib (11). Saijo et al. (12) observed catheter rupture in 3 (4.5%) of 66 patients who had a CV port placed via the internal jugular vein. Furthermore, in our previous study of 184 cases of CV port placement for advanced colorectal cancer, the CV port was placed through the internal jugular vein in 180 (98%) patients; of these, 5 (2.7%) experienced catheter injury, including 1 (0.5%) case of CV port rupture (13). Busch reported that the frequency of catheter rupture in 533 patients with CV ports placed in the upper arm was 1.8% (14). Thus, catheter rupture occurs regardless of which vein is used for CV port placement.

Previous studies have reported cases of catheter damage, both complete rupture and partial damage, in CV ports placed via multiple venous approaches. We found six cases of complete rupture of CV port catheters placed via the right internal jugular vein in the literature (12, 15–17) (**Table 2**). CV port placement via the internal jugular vein approach reportedly has a higher success rate and fewer complications than the subclavian vein approach (18). Additionally, recent reports have suggested the usefulness of CV port placement via the innominate vein or brachiocephalic vein (19, 20). While no conclusive evidence has been established regarding which approach is most preferable, we currently opt for ultrasound-guided CV port placement via the internal jugular vein.

**Table 2:** Reports of catheter rupture in the CV port device placed via the internal jugular vein.

Author	Age (years), sex	Primary site	Type of device^®^	Duration to rupture (month)	Symptom upon discovery	Location of distal catheter fragment	Removal method of catheter fragment
(Shimizu et al., (17))	35, M	Lung	BARD MRI port	26	Right neck pain	Right atrium	Catheter intervention
(Pignataro et al., (15))	41, M	Colon	BARD portacath	24	Right neck pain Subcutaneous swelling	Right atrium	Catheter intervention
(Ko et al., (16))	50, F	Ovary	DistricAth	3	Subcutaneous swelling	Right atrium	Catheter intervention
(Saijo et al., (12))	64, M 78, F 59, F	Prostate Breast Breast	PowerPort PowerPort PowerPort	21 31 38	Cough (caused by pneumonia) Catheter occlusion of CV port Catheter occlusion of CV port	Right atrium Inferior vena cava Right ventricle	Catheter intervention Catheter intervention Catheter intervention

CV, central venous; M, male; F, female.

We report four cases (0.6%) of catheter rupture among 726 patients who underwent CV port placement via the right internal jugular vein. The cause of catheter rupture was thought to be stress that weakened the catheter. Another possible reason was the use of the Groshong CV port. Groshong catheters, made of silicone, are considered more vulnerable than open-ended polyurethane catheters (21). Furthermore, the median duration of CV port implantation in the four patients was 39 months, which is relatively long. Thus, the risk of rupture should be considered when implanting a grossing-type CV port for a long period; the type of CV port needs to be considered carefully when it is required for long-term use.

Among the four patients with catheter rupture in the current series, in one patient, the rupture went unnoticed, and the ruptured CV port continued being used. The delay in detecting catheter rupture occurred because the ruptured part was distally located and remained in the intravascular region. Furthermore, the physicians in charge did not assess the position of the catheter tip in CT images. In fact, by the time the rupture was detected, 13 months had elapsed, and five CT scans were performed during this period. We were preoccupied with the tumor size and missed the catheter rupture. To our knowledge, while no study has reported serious complications from continued use of ruptured catheters and no serious complications occurred in our patients, imaging scans, even in asymptomatic cases, must be assessed for the possibility of catheter rupture to prevent complications. Further, the CV ports were not being used in three patients after initial therapy. Catheter rupture of the CV port is an infrequent complication associated with long-term placement of CV ports, and it can have serious sequela. Hence, the CV port must be removed when no longer required.

In all four cases with ruptured CV ports, the ruptured catheters were retrieved using an interventional technique, with no complications. Most patients with torn catheters are asymptomatic, but in rare cases, cardiac perforation, arrhythmias, and thromboembolism may occur; hence, their retrieval is advisable (22). A torn catheter can be retrieved by several methods, including interventional methods and surgical, open-chest approaches; the less invasive interventional approach is popular (6). In fact, intravascular catheterization has a retrieval rate of approximately 94–98% of torn catheters (23, 24).

In one of our patients in whom the torn catheter had remained in the right atrium for a long period, the catheter was removed without any granulation tissue, although we suspected that catheter removal would be difficult owing to adhesion to the surrounding tissue. The interventional technique is effective in retrieving torn catheters owing to the high probability of retrieval and low complication rate. Huang et al. (25) noted that while technical advancements have reduced complications associated with CV ports, international guidelines on this procedure are still lacking. They emphasize the need for multicenter prospective trials to validate optimal insertion vessels, catheter tip positioning, and catheter management during CV port placement.

This case series has several limitations. Our series included only four cases at a single institution. Although we mentioned the possible causes of catheter rupture, we have not been able to prove a definitive causal relationship. Further multicenter studies with a larger number of cases are, thus, needed.

## 4 Conclusion

Catheter rupture of the CV port is an infrequent complication, and breakage rates are expected to decrease as catheter materials improve. Nevertheless, since catheter rupture can lead to serious complications, routine monitoring is required, regardless of the vein into which they are inserted, and the possibility of catheter rupture should also be considered if the CV port is left *in situ* for an extended period.

## Data Availability

The raw data supporting the conclusions of this article will be made available by the authors, without undue reservation.
